# Rare-ID: Genomic Diagnosis in Symptomatic Neonates and Young Infants with Complex Clinical Phenotypes: A Descriptive Cohort Study

**DOI:** 10.3390/genes17080952

**Published:** 2026-08-14

**Authors:** Yannis L. Loukas, Katherine Anagnostopoulou, Georgia Thodi, Maria Spanou, Christos Gavalas, Elina Molou, Stefania Antonopoulou, Antigoni Poulopoulou, Yannis Dotsikas, Maria Alvanou, Konstantinos Tegopoulos, Roser Pons, Konstantinos Tziouvas, Georgios Vartzelis, Eleni Skouteli, Eirini Loukatou, Antonia Charitou, Konstantinos Douros, Soultana Siahanidou, Melpomene Giorgi, Artemis Stephanede, Maria Angeli, Maria Nikolaidou, Eleftheria Kokkinou, Ioanna Kouri, Vasiliki Koute, Eleni Frysira, Argirios Dinopoulos

**Affiliations:** 1 Section of Pharmaceutical Chemistry, Department of Pharmacy, National and Kapodistrian University of Athens, Zografou, 15771 Athens, Greece; idotsik@pharm.uoa.gr; 2 Neoscreen Molecular Diagnostic Laboratory, Vrilissia, 15235 Athens, Greece; kat_anag@yahoo.com (K.A.); c.gavalas@neoscreengr.com (C.G.); lab2@neoscreengr.com (E.M.); m.alvanou@neoscreengr.com (M.A.); 3 Neogenetics Molecular Diagnostic Laboratory, Vrilissia, 15235 Athens, Greece; lab@neoscreengr.com (G.T.); molbiol@neoscreengr.com (S.A.); molbiol1@neoscreengr.com (A.P.); 4 Pediatric Neurology Division, Third Department of Pediatrics, “Attikon” University Hospital, School of Medicine, National and Kapodistrian University of Athens, 12462 Athens, Greece; mspanou23@gmail.com (M.S.); mel.giorgi@hotmail.com (M.G.); artemis_stef@hotmail.com (A.S.); aggeli92@hotmail.com (M.A.); marianikolaidou.89@gmail.com (M.N.); argydino@gmail.com (A.D.); 5 Department of Molecular Biology and Genetics, School of Health Sciences, Democritus University of Thrace, University Campus, Dragana, 68100 Alexandroupolis, Greece; ktegopou@mbg.duth.gr; 6 First Department of Pediatrics, Aghia Sophia Children’s Hospital, School of Medicine, National and Kapodistrian University of Athens, 11527 Athens, Greece; roserpons@med.uoa.gr (R.P.); el_kok@otenet.gr (E.K.); 7 Pediatric Intensive Care Unit, P. & A. Kyriakou Children’s Hospital, 11527 Athens, Greece; ktziouvas@yahoo.com; 8 Department of Neurology, P. & A. Kyriakou Children’s Hospital, 11527 Athens, Greece; vartzelius@yahoo.gr; 9 Pediatric Neurology, ELEPAP—Rehabilitation for the Disabled, 11634 Athens, Greece; hskouteli@yahoo.co.uk; 10 Pediatric Neurology, MITERA Children’s Hospital, 15123 Athens, Greece; ioankouri@gmail.com; 11 Department of Pediatrics, Alexandra General Hospital, 11528 Athens, Greece; neogn.dir@hosp-alexandra.gr; 12 Department of Pediatrics, REA Maternity Hospital, Palaio Faliro, 17564 Athens, Greece; antoniacharitou@gmail.com; 13 Pediatric Allergy and Respiratory Unit, Third Department of Pediatrics, “Attikon” University Hospital, School of Medicine, National and Kapodistrian University of Athens, 12462 Athens, Greece; costasdouros@gmail.com; 14 Neonatal Unit, First Department of Pediatrics, Aghia Sophia Children’s Hospital, School of Medicine, National and Kapodistrian University of Athens, 11527 Athens, Greece; siahan@med.uoa.gr; 15 Department of Pediatrics, University General Hospital of Larissa, Faculty of Medicine, University of Thessaly, 41110 Larissa, Greece; koutevas@yahoo.gr; 16 Laboratory of Medical Genetics, Choremio Research Laboratory, Aghia Sophia Children’s Hospital, School of Medicine, National and Kapodistrian University of Athens, 11527 Athens, Greece; efrysira@gmail.com

**Keywords:** genomic diagnosis, whole-exome sequencing, whole-genome sequencing, symptomatic neonates, young infants, rare disease, clinical utility

## Abstract

**Background/Objectives:** Genomic sequencing can shorten the diagnostic pathway for selected symptomatic neonates and young infants, but evidence from such cohorts should not be extrapolated to population newborn screening. This study describes molecular findings and potential clinical implications in 25 unrelated patients younger than 6 months at referral with heterogeneous, predominantly neurological phenotypes and no established molecular diagnosis. **Methods:** The first 17 patients underwent whole-exome sequencing (WES), and the subsequent 8 underwent whole-genome sequencing (WGS) under sequential laboratory protocols; allocation was not randomized, and the study was not designed to compare platforms. **Results:** Pathogenic or likely pathogenic findings providing a definitive or likely molecular diagnosis were identified in 7/25 patients (28.0%; 95% confidence interval [CI], 14.3–47.6), including sequence variants, one 20q13.33 deletion, and mosaic trisomy 9. An additional RANBP2 variant was interpreted as a susceptibility-associated finding in a patient with infection-related encephalitis, yielding clinically relevant findings in 8/25 patients (32.0%; 95% CI, 17.2–51.6). Three definitive diagnoses involved disorders with established disease-specific management considerations; however, patient-level treatment changes, turnaround times, and outcomes were not systematically assessed. **Conclusions:** These findings support the diagnostic value of genomic testing in selected symptomatic neonates and young infants, while the small, heterogeneous cohort, sequential non-equivalent workflows, and incomplete outcome data preclude conclusions about comparative WES/WGS performance or population newborn screening.

## 1. Introduction

Next-generation sequencing (NGS) has transformed the diagnosis of rare disease by allowing simultaneous evaluation of many genes rather than sequential single-gene testing. Following the development of massively parallel sequencing and early demonstrations of exome-based disease-gene identification, WES and WGS have become established tools in undiagnosed disease and neurodevelopmental medicine [1,2,3,4,5,6,7,8,9,10,11,12,13,14,15,16,17,18]. Their clinical value is greatest when the result is interpreted in the context of a well-defined phenotype, family history, inheritance model, and the patient’s stage of illness.

WES primarily interrogates coding regions, whereas WGS provides more uniform genome-wide coverage and can support analysis of a broader range of variant classes. Nevertheless, analytic scope depends not only on the sequencing platform but also on library preparation, coverage, bioinformatic tools, validation, and reporting policy [19,20,21,22,23,24,25,26,27,28,29,30,31,32]. Exome data can also support copy-number analysis when a validated caller and appropriate reference framework are used; therefore, detection of a structural finding in a WGS subgroup does not by itself establish platform superiority.

Published diagnostic yields vary substantially according to clinical indication, acuity, previous testing, singleton versus trio analysis, and the variant classes included [17,33,34,35,36,37,38,39,40,41,42,43,44,45]. Consequently, yield estimates from a small clinically referred cohort should be interpreted descriptively rather than as universal benchmarks.

Phenotype-driven genomic diagnosis in symptomatic neonates or infants is conceptually different from population newborn screening. Diagnostic testing begins with a selected patient who already has clinical findings and a higher pre-test probability of genetic disease. Population newborn screening is applied to an unselected, largely presymptomatic population and requires separate evidence regarding sensitivity, specificity, positive predictive value, penetrance, actionability, consent, follow-up pathways, equity, and program feasibility [46,47].

The aim of the Rare-ID study was therefore to describe the spectrum of molecular findings obtained using sequential WES and WGS workflows in 25 symptomatic neonates and young infants without an established diagnosis, and to characterize the potential clinical implications of those findings. The study was not designed as a randomized or paired comparison of WES and WGS and does not evaluate population newborn screening.

## 2. Materials and Methods

### 2.1. Study Design, Participants, and Ethics

This descriptive observational cohort included 25 unrelated symptomatic neonates and young infants younger than 6 months at referral/testing. Patients were referred from neonatal and pediatric services across Greece between January 2019 and December 2022 because a genetic disorder was suspected and no molecular diagnosis had been established. Referral indications were heterogeneous and included seizures or epileptic encephalopathy, hypotonia or neuromuscular weakness, congenital anomalies or dysmorphism, metabolic or multisystem abnormalities, respiratory/autonomic presentations, and acquired or potentially acquired encephalopathy. Patients with malignant tumors and patients whose legal representatives could not provide informed consent were excluded.

The median recorded age at symptom onset was 2.5 days (interquartile range [IQR], birth to 11.5 days; n = 24 with known onset), and the median age at referral/testing was approximately 1 month (IQR, 24–58 days; range, 4 days to 6 months). At least one neurological feature was recorded in 21/25 patients (84%). Complete sex, gestational-age, prematurity, and care-setting data were not consistently available in the analytical dataset and are therefore not summarized.

Peripheral-blood samples were obtained from probands and, when available for targeted segregation testing, from parents. Written informed consent was obtained from legal representatives. The study was approved by the ethics committees of Attikon University General Hospital (G7, 7 October 2021) and the Department of Pharmacy, National and Kapodistrian University of Athens (1277, 25 May 2020).

### 2.2. Clinical Phenotyping and Descriptive Categories

Referring physicians supplied narrative phenotype information and family history, with neuroimaging, EEG, metabolic testing, and other investigations provided when available. Formal prospective Human Phenotype Ontology (HPO) coding was not documented for this cohort. For the present analysis, recorded presentations were retrospectively organized into six mutually exclusive primary categories: (i) primary neurological/epileptic, (ii) hypotonia/neuromuscular, (iii) congenital anomalies/dysmorphism/growth, (iv) metabolic or hematologic/multisystem, (v) acquired or mixed-acquired encephalopathy, and (vi) respiratory/autonomic. These categories were used only for descriptive stratification and not for inferential testing. The individual clinical characteristics and sequencing strategies are summarized in Table 1.

### 2.3. DNA Extraction, Sequencing, and Allocation of WES and WGS

Genomic DNA was extracted from peripheral blood using the NucleoSpin Tissue kit (MACHEREY-NAGEL GmbH & Co. KG, Düren, Germany). Sequencing was performed by BGI Genomics Co., Ltd. (Shenzhen, China) on a DNBSEQ-T7 platform (MGI Tech Co., Ltd., Shenzhen, China) with 150-bp paired-end reads. The first 17 enrolled probands (Patients 1–17) were analyzed by WES under the laboratory protocol in use at that stage of the project. After implementation of a PCR-free WGS workflow, the subsequent 8 probands (Patients 18–25) were analyzed by WGS. Allocation was therefore chronological and non-randomized; the groups are not contemporaneous comparator arms.

WES achieved a reported average depth greater than 100×, and WGS achieved a reported average depth greater than 30×. The analytical dataset used for this report contained cohort-level average depth but not complete patient-level exon breadth, phenotype-relevant low-coverage intervals, or resequencing logs. Accordingly, average depth is not interpreted as evidence that every clinically relevant exon met a predefined coverage threshold, and negative WES results retain a residual false-negative risk.

### 2.4. Bioinformatic Analysis

For WES, germline SNVs and small insertions/deletions were called using Genome Analysis Toolkit (GATK) v4.4 and DeepVariant v1.6 [25,26]. Annotation and in silico predictions incorporated dbNSFP v4.0, including SIFT, LRT, MutationTaster, FATHMM, PROVEAN, MetaSVM, MetaLR, MetaRNN, M-CAP, PrimateAI, DEOGEN2, BayesDel, and LIST-S2 [27]. A dedicated, validated exome-CNV caller was not documented in the WES workflow reported here; therefore, the study does not claim equivalent CNV sensitivity for WES and WGS.

For WGS, SNVs and small insertions/deletions were analyzed using the same GATK/DeepVariant strategy [25,26,28]. WGS data were additionally evaluated through the laboratory’s proprietary three-algorithm workflow for copy-number and chromosomal abnormalities. Algorithm-specific names, versions, thresholds, and validated mosaic sensitivity were not available in the retrospective analytical dataset, which limits reproducibility and precludes benchmarking of structural-variant performance.

### 2.5. Variant Interpretation, Family Testing, and Validation

Annotated variants were reviewed in the NsClinical–Neoscreen Genomics App (the software version used during the study was not retained in the retrospective dataset) and interpreted using the 2015 American College of Medical Genetics and Genomics/Association for Molecular Pathology (ACMG/AMP) framework, ClinVar assertions, inheritance information when available, and phenotype–genotype concordance [48]. Only pathogenic or likely pathogenic variants, pathogenic chromosomal findings, and the separately designated RANBP2 susceptibility-associated finding were included in the present results; variants of uncertain significance were not counted as diagnoses. Systematic periodic reanalysis of all negative cases was outside the scope of this report.

The core sequencing design was proband-based rather than a systematic trio design. Targeted parental testing was performed in six diagnosed cases (Patients 1, 2, 3, 8, 17, and 21) to assess segregation or de novo status. The six SNV/small-indel findings were confirmed by Sanger sequencing on an ABI 3130xl Genetic Analyzer (Applied Biosystems, Foster City, CA, USA) using primers designed with Primer3 v0.4.0 [29]. The original blanket statement that all variant classes were confirmed by Sanger sequencing was incorrect and has been removed. The available study dataset did not specify the independent cytogenetic assay used, if any, for the 20q13.33 deletion or mosaic trisomy 9; these are therefore reported as WGS-derived clinical laboratory findings, and the absence of assay-level orthogonal-confirmation details is acknowledged as a limitation.

### 2.6. Statistical Analysis

Analyses were descriptive. Ages expressed in months were converted to days using 30.44 days per month for summary statistics. Diagnostic proportions are presented with two-sided 95% Wilson confidence intervals. WES and WGS subgroup yields are reported for transparency only; no hypothesis test or comparative effect estimate was performed because platform allocation was sequential, sample sizes were small, and the analytic scopes were not equivalent.

## 3. Results

### 3.1. Cohort and Phenotype Distribution

The cohort was clinically heterogeneous, although neurological involvement predominated. The six descriptive primary categories comprised 9 patients with a primary neurological/epileptic presentation, 5 with a hypotonia/neuromuscular presentation, 4 with congenital anomalies/dysmorphism/growth abnormalities, 2 with metabolic or hematologic/multisystem presentations, 3 with acquired or mixed-acquired encephalopathy, and 2 with respiratory/autonomic presentations. Because category denominators were small, category-specific yields are reported as case counts rather than generalizable estimates.

### 3.2. Molecular Findings and Diagnostic Yield

Seven patients had a pathogenic, likely pathogenic, or chromosomal finding considered to provide a definitive or likely molecular diagnosis, corresponding to a diagnostic yield of 28.0% (7/25; 95% CI, 14.3–47.6). These findings comprised five SNV/small-indel diagnoses, one 20q13.33 deletion, and one mosaic trisomy 9. Patient 9 carried the RANBP2 c.1966A>G, p.(Ile656Val) variant in the setting of COVID-19-associated encephalitis. Because RANBP2 variants confer incompletely penetrant susceptibility to infection-triggered acute encephalopathy rather than a fully penetrant standalone disorder, this finding was reported separately and was not included in the definitive diagnostic numerator [49]. Including this susceptibility-associated finding, clinically relevant findings were reported in 8/25 patients (32.0%; 95% CI, 17.2–51.6). The individual molecular findings are summarized in Table 2.

Among the WES cases, 5/17 had a definitive diagnosis (29.4%; 95% CI, 13.3–53.1), and 6/17 had a clinically relevant finding when the RANBP2 susceptibility result was included (35.3%; 95% CI, 17.3–58.7). Among the WGS cases, 2/8 had a definitive diagnosis (25.0%; 95% CI, 7.1–59.1). These observed proportions are descriptive and are not a valid head-to-head comparison. The phenotype-category findings are summarized in Table 3.

Targeted parental testing indicated that the KCNQ2 frameshift in Patient 17 and the 20q13.33 deletion in Patient 21 were de novo. The parents of Patients 1, 2, 3, and 8 were heterozygous carriers of the corresponding autosomal-recessive variants. Among the six definitive sequence/CNV diagnoses, four were autosomal recessive and two were de novo autosomal dominant; the seventh definitive finding was mosaic trisomy 9.

### 3.3. Clinical Implications and Available Outcomes

Genetic counseling was provided to the families of diagnosed patients. Three of the seven definitive diagnoses (MOCS1, ECHS1, and KCNQ2) involved disorders with established disease-specific management considerations (3/7; 42.9%; 95% CI, 15.8–75.0). This proportion represents potential actionability, not a measured clinical-utility endpoint. Complete patient-level data on sample-to-report turnaround time, whether results preceded acute decisions, actual treatment changes, treatment response, and long-term outcomes were not available for all participants. Accordingly, the revised manuscript does not claim that sequencing improved outcomes or family quality of life. The clinical implications and available outcome information are summarized in Table 4.

Patient 1 had a homozygous EXOSC3 diagnosis consistent with pontocerebellar hypoplasia type 1B in the context of congenital hypotonia, respiratory failure, and multiple infant deaths in the family [50]. No targeted therapy is available; the infant died in infancy, while the diagnosis provided recurrence-risk clarification and reproductive options. Patient 8 had a homozygous ECHS1 diagnosis, for which valine-restricted dietary management may be considered [51,52], although the timing and clinical response were not available in the analyzed dataset. Patient 23 had mosaic trisomy 9, a diagnosis relevant to prognosis, multisystem surveillance, and counseling [53,54].

## 4. Discussion

In this descriptive cohort of 25 symptomatic neonates and young infants, 7 patients had a definitive or likely molecular diagnosis and one additional patient had an RANBP2 susceptibility-associated finding. The wide 95% confidence intervals reflect the small sample and emphasize that the observed 28.0% diagnostic yield is a cohort-specific estimate. The referral population was heterogeneous, with different prior probabilities of a monogenic disorder; therefore, the overall yield cannot be generalized to all NICU patients, all infants with neurological symptoms, or an unselected newborn population.

The WES and WGS groups were sequential, non-randomized, and analytically non-equivalent. The WGS workflow identified a 20q13.33 deletion and mosaic trisomy 9, but this observation cannot establish superior WGS performance because the WES workflow did not document a dedicated validated CNV caller and the groups differed by calendar period. Tools such as CNVkit demonstrate that multi-exon and larger CNVs can be inferred from targeted sequencing data when an appropriate reference set, normalization, validation, and reporting framework are used [55]. Genome-wide sequencing has also been used to characterize heterogeneous fetal central nervous system anomalies [56]. A valid platform comparison would require paired WES/WGS on the same patients or contemporaneous allocation with equivalent variant classes, quality thresholds, orthogonal confirmation, and blinded interpretation.

To answer the incremental performance question directly, future work should use paired same-patient analysis. The strongest design would sequence the same DNA by WES and WGS, preferably in trios, and apply blinded interpretation with matched variant classes, quality thresholds, orthogonal confirmation, and prespecified endpoints. A pragmatic clinical design could use WES followed by reflex WGS in unresolved cases to estimate the additional yield after WES; alternatively, WGS data could be analyzed first as a restricted virtual exome and then genome-wide, although this would not reproduce exome-capture limitations. Such studies should measure incremental diagnostic yield, time to diagnosis, cost, and management impact [30].

The distinction between definitive diagnosis and susceptibility is also important. The RANBP2 p.(Ile656Val) variant has condition-specific pathogenic submissions for susceptibility to infection-induced acute encephalopathy, but aggregate ClinVar classifications are conflicting and penetrance is incomplete. In Patient 9, the finding may explain predisposition in the context of infection, but it is not equivalent to a fully penetrant Mendelian diagnosis such as EXOSC3-, MOCS1-, ECHS1-, or KCNQ2-related disease. Reporting it separately reduces inflation of the definitive diagnostic yield and better reflects the clinical uncertainty.

Several findings had potential management relevance. Very early recognition of MOCS1-related molybdenum cofactor deficiency can permit consideration of cyclic pyranopterin monophosphate replacement [57]. ECHS1 deficiency may prompt valine-restricted dietary management and metabolic support [51,52], and KCNQ2-related epilepsy can inform antiseizure-drug selection [58]. Nevertheless, potential actionability is not the same as demonstrated clinical utility. Because complete turnaround-time, treatment-change, and outcome data were unavailable, this study cannot determine whether genomic results improved acute or long-term outcomes. Counseling, recurrence-risk clarification, and avoidance of further diagnostic testing remain meaningful forms of utility, but quality of life was not formally measured.

Importantly, a negative initial result should not be equated with exclusion of genetic disease. For the 18 patients without a definitive diagnosis, a future structured pathway would include clinical re-phenotyping and HPO coding; trio-based reinterpretation; periodic reanalysis as gene-disease and variant knowledge evolves; explicit assessment of CNVs, structural variants, mosaicism, mitochondrial variants, and repeat expansions; and, when phenotype-directed, RNA sequencing, methylation/episignature testing, long-read sequencing, metabolomic/proteomic studies, or functional validation. Data sharing and tissue-specific testing may be appropriate in selected cases, while acquired and multifactorial diagnoses should remain in the differential. These approaches were not systematically undertaken in the present study and are proposed as future strategies rather than reported results [59,60].

Existing professional guidance supports consideration of exome/genome sequencing as a first- or second-tier test for selected pediatric patients with congenital anomalies or developmental delay, but this is not equivalent to a universal management protocol for every genomic finding [61]. In the present cohort, no single protocol governed all detected conditions. Post-test management should be diagnosis-specific and should consider disease-gene validity, variant certainty, penetrance, age of onset, severity, and the evidence that presymptomatic surveillance or treatment improves outcomes. A balanced pathway is neither to wait invariably for irreversible phenotype nor to intervene solely on genotype: results should be confirmed, disclosed with genetic counseling, followed by baseline assessment and relevant specialist referral, and translated into surveillance or treatment only when the expected benefit exceeds burden and risk. Conditions lacking established neonatal guidance require multidisciplinary review, shared decision-making, and prospective outcome collection.

An apparently healthy newborn is not necessarily unaffected. Some disorders are presymptomatic at birth, and screening may reveal subclinical or age-dependent disease risk. Such findings should often be framed as risk stratification rather than immediate diagnosis. Reliable penetrance estimates require representative population-based ascertainment, standardized baseline phenotyping, confirmation of positive findings, and longitudinal follow-up of both carriers and noncarriers; cross-sectional absence of symptoms at birth is insufficient. Newborn sequencing studies such as BabySeq illustrate both the possibility of unanticipated actionable findings and the interpretive burden created by variable penetrance and downstream follow-up [62,63]. The present symptom-enriched cohort cannot estimate penetrance, positive predictive value, or false-positive burden in apparently healthy newborns.

Published economic analyses suggest that early exome or genome sequencing may be cost-effective in selected pediatric diagnostic pathways [10,64,65,66,67]. The present study did not collect costs, resource utilization, or comparative diagnostic-trajectory data, and therefore provides no direct cost-effectiveness estimate. Likewise, peripheral blood was used in this cohort, but no comparison with dried blood spots was performed; specimen type and workflow performance should not be inferred from these data [46].

This study has several limitations: the cohort was small and clinically heterogeneous; referral was not designed to produce a population-representative sample; WES and WGS were allocated sequentially; variant-class analysis was not equivalent across workflows; systematic trio sequencing was not performed; formal prospective HPO phenotyping was not documented; patient-level coverage breadth and resequencing data were incomplete; algorithm-level details for the proprietary WGS structural-variant workflow were unavailable; orthogonal confirmation details for the deletion and mosaic aneuploidy were not available for reporting; negative cases were not systematically reanalyzed; and turnaround time, treatment changes, costs, and long-term outcomes were not systematically assessed. These limitations prevent comparative performance, causal clinical-utility, cost-effectiveness, and population-screening conclusions. The study also lacked a paired same-patient platform comparison and a prespecified post-negative diagnostic escalation pathway.

## 5. Conclusions

These findings support the diagnostic value of genomic sequencing in selected symptomatic neonates and young infants with complex, mainly neurological phenotypes. The study also illustrates that clinically relevant results may include sequence variants, copy-number changes, chromosomal abnormalities, and susceptibility-associated findings that require different levels of interpretive certainty. However, the small sample size, clinical heterogeneity, sequential non-uniform use of WES and WGS, and incomplete technical and outcome data limit generalizability. Larger prospective studies using standardized phenotyping, uniform and validated variant-class analysis, trio-based sequencing where appropriate, predefined turnaround-time and clinical-utility endpoints, orthogonal confirmation, and long-term outcome assessment are required before extrapolating diagnostic findings from symptomatic infants to population newborn screening. Future work should include paired same-patient WES/WGS designs, structured reanalysis and phenotype-directed second-tier testing for unresolved cases, explicit disease-specific post-test pathways, and prospective population-based newborn cohorts with long-term follow-up to estimate age-specific penetrance and predictive value.

## Figures and Tables

**Table 1:** Clinical characteristics and sequencing strategy for the 25 participants.

Patient	Test	Recorded Clinical Phenotype	Age at Onset	Age at Referral/Testing
1	WES	Hypotonia; respiratory distress	At birth	24 d
2	WES	Hypotonia; enlarged cisterna magna/cerebellomedullary cistern; short neck; livedo reticularis	20 d	1.5 mo
3	WES	Microcephaly; hypodontia; afebrile seizures; dysmorphic facial features; severe developmental impairment; epileptic encephalopathy	10 d	1 mo
4	WES	Metabolic acidosis; hypotonia; vomiting; diarrhea; endocrine and intermediary-metabolism abnormalities	2 d	1 mo
5	WES	Head and neck tremor	5.5 mo	6 mo
6	WES	Neonatal seizures; epileptic encephalopathy	At birth	8 d
7	WES	Truncal hypotonia; ventriculomegaly; limb hypertonia; head lag; corpus-callosum and ventricular MRI abnormalities	At birth	24 d
8	WES	Infantile spasms; epilepsy; leukoencephalopathy; MRI and EEG abnormalities; sensorineural hearing loss; feeding difficulties	4 d	42 d
9	WES	COVID-19-associated encephalitis	5 d	7 d
10	WES	Lissencephaly	Unknown	3.5 mo
11	WES	Lower-limb hypoplasia/skeletal abnormalities; varus forefoot; cryptorchidism	At birth	4 d
12	WES	Acute life-threatening event; acute encephalopathy; seizures; hypoglycemia; cytotoxic edema affecting cerebral hemispheres and cerebellum	5 mo	6 mo
13	WES	Low birth weight; seizures; limb hypertonia; head lag; dysmorphic features; basal-ganglia/white-matter MRI abnormalities; poor growth; respiratory failure	At birth	6 mo
14	WES	Extremely low birth weight; intrauterine growth restriction; dysmorphic features; saddle-nose deformity	At birth	1 mo
15	WES	Neonatal seizures	2 d	22 d
16	WES	Apnea; bradycardia; cyanosis	1 mo	2 mo
17	WES	Infantile spasms	3 d	26 d
18	WGS	Hypotonia; muscle weakness; micrognathia; congenital lower-limb abnormalities; respiratory failure; feeding difficulties; jaundice; clubfoot; knee hyperextension; MRI abnormalities	At birth	12 d
19	WGS	Feeding difficulties; truncal hypotonia; weight loss	9 d	19 d
20	WGS	Tachypnea; prolonged expiration; wheezing	2 d	57 d
21	WGS	Infantile spasms	3 d	40 d
22	WGS	Focal seizures; hypotonia; epileptic encephalopathy; basal-ganglia MRI abnormalities; EEG abnormalities	16 d	25 d
23	WGS	Prematurity; neurodevelopmental delay; Duane retraction syndrome type III; reduced eye contact and communication	5 mo	5 mo
24	WGS	Jaundice; thrombocytopenia; anemia; hepatosplenomegaly; grade 2–3 intraventricular hemorrhage	At birth	30 d
25	WGS	Hypoxic–ischemic encephalopathy; MRI and EEG abnormalities; seizures; hypertonia; absent suck reflex; no congenital anomalies	At birth	58 d

d: days; mo: months; EEG: electroencephalography; MRI: magnetic resonance imaging; WES: whole-exome sequencing; WGS: whole-genome sequencing. “Unknown” indicates that a reliable onset age was not available.

**Table 2:** Clinically relevant molecular findings and interpretive category.

Patient	Test	Gene/Variant or Chromosomal Finding	Inheritance	Classification and Study Interpretation	Associated Condition
1	WES	EXOSC3 c.92G>C, p.(Gly31Ala), homozygous	AR; inherited	Pathogenic; definitive diagnosis	Pontocerebellar hypoplasia type 1B
2	WES	EXOSC3 c.92G>C, p.(Gly31Ala), homozygous	AR; inherited	Pathogenic; definitive diagnosis	Pontocerebellar hypoplasia type 1B
3	WES	MOCS1 c.1508_1509del, p.(Glu503Alafs*103), homozygous	AR; inherited	Likely pathogenic; definitive diagnosis	Molybdenum cofactor deficiency type A
8	WES	ECHS1 c.476A>G, p.(Gln159Arg), homozygous	AR; inherited	Pathogenic; definitive diagnosis	ECHS1-related mitochondrial short-chain enoyl-CoA hydratase deficiency
9	WES	RANBP2 c.1966A>G, p.(Ile656Val), heterozygous	AD susceptibility; inheritance not established	Condition-specific pathogenic submissions; susceptibility-associated finding	Susceptibility to infection-induced acute encephalopathy; not counted as a definitive diagnosis
17	WES	KCNQ2 c.1229dupC, p.(Pro411Alafs*7), heterozygous	AD; de novo	Pathogenic; definitive diagnosis	KCNQ2-related developmental and epileptic encephalopathy
21	WGS	20q13.33 deletion, GRCh38 chr20:62,751,315-63,689,630, approximately 938 kb	De novo	Pathogenic; definitive diagnosis	20q13.33 deletion involving COL9A3, SLC17A9, CHRNA4, KCNQ2, EEF1A2, and RTEL1
23	WGS	Mosaic trisomy 9, approximately 30% in peripheral-blood WGS data	Chromosomal	Pathogenic; definitive diagnosis	Trisomy 9 mosaicism

AD: autosomal dominant; AR: autosomal recessive. The RANBP2 finding is reported as susceptibility-associated and is excluded from the 7/25 definitive diagnostic yield. De novo/inherited status reflects targeted parental testing where available. An asterisk (*) in protein-level HGVS notation denotes a termination codon.

**Table 3:** Descriptive findings by primary phenotype category.

Primary Phenotype Category	Patients, n	Definitive Diagnoses, n/N (%)	Additional Susceptibility Finding
Primary neurological/epileptic phenotype	9	4/9 (44.4%)	None
Hypotonia/neuromuscular phenotype	5	2/5 (40.0%)	None
Congenital anomalies/dysmorphism/growth phenotype	4	1/4 (25.0%)	None
Metabolic or hematologic/multisystem phenotype	2	0/2	None
Acquired or mixed-acquired encephalopathy	3	0/3	1 RANBP2 susceptibility-associated finding
Respiratory/autonomic presentation	2	0/2	None

Categories were assigned retrospectively from the recorded referral phenotype. Percentages are descriptive and should not be generalized because denominators are very small and patients had overlapping features.

**Table 4:** Type of clinical implication and availability of outcome information for clinically relevant findings.

Patient/Finding	Documented Diagnostic or Counseling Consequence	Disease-Specific Management Consideration	Outcome Information Available for This Report
1, EXOSC3	Etiologic diagnosis, prognosis, recurrence-risk counseling, and reproductive options	Supportive respiratory/nutritional care; no disease-specific therapy	Infant died in infancy; detailed timing and follow-up were not available
2, EXOSC3	Etiologic diagnosis and reproductive counseling	Supportive care	Outcome not available in the analyzed dataset
3, MOCS1	Etiologic diagnosis and counseling	cPMP replacement is time-sensitive in MOCS1-related deficiency	Treatment initiation, timing, and outcome were not available
8, ECHS1	Etiologic diagnosis and counseling	Valine-restricted dietary management and metabolic support may be considered	Actual dietary change and response were not available
9, RANBP2	Susceptibility information and family counseling	Infection-trigger awareness and early evaluation during febrile illness	Outcome and recurrence data were not available
17, KCNQ2	Etiologic diagnosis and counseling	Antiseizure-drug selection may be informed by KCNQ2 genotype	Medication change and seizure response were not available
21, 20q13.33 deletion	Syndromic diagnosis, prognosis, surveillance planning, and counseling	Multidisciplinary neurologic and developmental follow-up	Outcome not available in the analyzed dataset
23, mosaic trisomy 9	Chromosomal diagnosis, prognosis, organ-system surveillance, and counseling	Multidisciplinary follow-up based on affected systems	Outcome not available in the analyzed dataset

The table distinguishes documented diagnostic/counseling consequences from disease-specific management possibilities. It does not imply that the listed intervention was initiated or that an outcome benefit occurred.

## Data Availability

De-identified data supporting the reported aggregate findings may be available from the corresponding author upon reasonable request and subject to ethics, consent, and privacy restrictions.

## Abbreviations

ACMG/AMP	American College of Medical Genetics and Genomics/Association for Molecular Pathology
AD	autosomal dominant
AR	autosomal recessive
CI	confidence interval
CNV	copy-number variant
EEG	electroencephalography
HPO	Human Phenotype Ontology
IQR	interquartile range
MRI	magnetic resonance imaging
NGS	next-generation sequencing
SNV	single-nucleotide variant
VUS	variant of uncertain significance
WES	whole-exome sequencing
WGS	whole-genome sequencing
